# Pharmacokinetics of Free Oxytetracycline and Oxytetracycline Loaded Cockle Shell Calcium Carbonate-Based Nanoparticle in *BALB/c* Mice

**DOI:** 10.3389/fvets.2020.00270

**Published:** 2020-06-16

**Authors:** Sherifat Banke Idris, Arifah Abdul Kadir, Jesse F. F. Abdullah, Siti-Zubaidah Ramanoon, Muhammad Abdul Basit, Md Zuki Z. A. Abubakar

**Affiliations:** ^1^Department of Veterinary Preclinical Studies, Faculty of Veterinary Medicine, Universiti Putra Malaysia, Serdang, Malaysia; ^2^Department of Veterinary Pharmacology and Toxicology, Faculty of Veterinary Medicine, Usmanu Danfodiyo University, Sokoto, Nigeria; ^3^Department of Veterinary Clinical Studies, Faculty of Veterinary Medicine, Universiti Putra Malaysia, Serdang, Malaysia; ^4^Department of Farm and Exotic Animal Medicine and Surgery, Faculty of Veterinary Medicine, Universiti Putra Malaysia, Serdang, Malaysia; ^5^Department of Biosciences, Faculty of Veterinary Sciences, Bahauddin Zakariya University, Multan, Pakistan

**Keywords:** oxytetracycline, pharmacokinetics, *BALB/c* mice, calcium carbonate nanoparticle, HPLC

## Abstract

The development and utilization of nano-antibiotics is currently gaining attention as a possible solution to antibiotic resistance. The aim of this study was therefore to determine the pharmacokinetics of free oxytetracycline (OTC) and oxytetracycline loaded cockle shell calcium carbonate-based nanoparticle (OTC-CNP) after a single dose of intraperitoneal (IP) administration in *BALB/*c mice. A total of 100 female *BALB/c* mice divided into two groups of equal number (*n* = 50) were administered with 10 mg/kg OTC and OTC-CNP, respectively. Blood samples were collected before and post-administration from both groups at time 0, 5, 10, 15, and 30 min and 1, 2, 6, 24, and 48 h, and OTC plasma concentration was quantified using a validated HPLC-UV method. The pharmacokinetic parameters were analyzed using a non-compartment model. The *C*_max_ values of OTC in OTC-CNP and free OTC treated group were 64.99 and 23.53 μg/ml, respectively. OTC was detected up to 24 h in the OTC-CNP group as against 1 h in the free OTC group following intraperitoneal administration. In the OTC-CNP group, the plasma elimination rate of OTC was slower while the half-life, the area under the curve, and the volume of the distribution were increased. In conclusion, the pharmacokinetic profile of OTC in the OTC-CNP group differs significantly from that of free OTC. However, further studies are necessary to determine the antibacterial efficacy of OTC-CNP for the treatment of bacterial diseases.

## Introduction

Oxytetracycline (OTC) is one of the frequently used antibiotics in livestock production (1). Its broad spectrum of activity and low cost compared to other antibiotics favor its use among veterinarians. However, this widespread use and misuse has resulted in resistance of bacterial pathogens to OTC (2). Recently, newer antibiotics have been favored over OTC in the treatment of infections in animals, but OTC is still used non-therapeutically as a growth promoter (3). Bacteria develop resistance to OTC through efflux pumps, ribosomal modification to reduce effective OTC binding, and the production of tetracycline inactivating enzymes (3, 4). An approach that can be used to solve this problem is the development of a nano-antibiotic delivery system. Nano-antibiotics delivery systems improve the pharmacokinetics and therapeutics and are able to bypass bacteria resistance mechanisms (5). Importantly, previous studies have shown that tetracyclines could be stably loaded and released from calcium-based nanoparticles (4, 6, 7) and also overcome the efflux pump antibiotic resistance mechanism of *Shigella flexineri* when loaded into calcium phosphate nanoparticles (CNPs) (4). The use of calcium-based nanoparticles is increasing not only due to their biodegradable and biocompatible properties but also because they can be engineered to stably load and release drugs within them in response to pH (7, 8). Calcium carbonate nanoparticles have unique liquid phase characteristics that enable them to be crystalline (stable) solids at pH 7.4 and disintegrate to form biocompatible non-toxic ions at lower pH (9). This property has been exploited to fabricate drug carriers in conditions where reduced pH is important such as the micro acidic environments created by biofilms, a major resistance mechanism, in chronic bacterial disease conditions (10, 11). The lower pH of the microenvironment within the biofilm extra polysaccharide matrix is due to anaerobic glycolysis and ion transfer challenges favoring the acidic medium within it (7, 12).

We hypothesized that loading OTC into a calcium carbonate aragonite nanoparticle (OTC-CNP) would improve its pharmacokinetics in *BALB/*c mice plasma compared to free OTC. To test this theory, we investigated the pharmacokinetics of 10 mg/kg of OTC-CNP and free OTC in female *BALB*/c mice.

## Materials and Methods

### Experimental Animals

A total of 100 female *BALB/c* mice were used in this study. They were housed in plastic cages with saw dust beddings, and clean tap water and a standard pellets diet (Gold coin mouse) were provided for the mice *ad libitum* throughout the time of the experiment. The mice were acclimatized for 1 week prior to the experiment. All procedures were done according to the research ethics of the Institutional Animal Care and Use Committee (IACUC) (UPM/IACUC/AUP/R050/2018).

### Study Design

One hundred female *BALB/c* mice were divided randomly into two groups of 50 mice each. Group 1 was administered with 10 mg/kg OTC intraperitoneally, while group 2 was dosed with 10 mg/kg OTC-CNP intraperitoneally. Briefly, 10 mg OTC was dissolved in 1 ml sterile distilled water, while 10 mg of freshly prepared OTC-CNP was dissolved in 1 ml sterile PBS (pH 7.4) to get the stock solution of 10 mg/ml. Then the weight of each mice was measured to get the calculated dose per mice in milligrams and the equivalent dose in milliliters (13). The choice of intraperitoneal route of administration for the pharmacokinetics of OTC in this study is justifiable because drug-nanoparticle formulations administrated via intraperitoneal injection increase the mean residence time of the drug in the peritoneal cavity, which improves systemic absorption (14). Also, the primary route of absorption for the IP route is through the mesenteric vessels, which drain into the portal veins and pass through the liver. Hence, this route could also be used to predict the oral bioavailability indirectly (15).

At specified times of 0, 5, 10, 15, and 30 min and 1, 2, 6, 24, and 48 h, five mice from each group were sacrificed after anesthesia with ketamine (80 mg/kg) and xylazine (10 mg/kg) cocktail. Blood was collected via cardiac puncture into heparinized tubes and centrifuged at 10,000 × g for 10 min to collect plasma. The plasma was then aliquoted to sterile small centrifuge tubes, labeled and frozen at −20°C until analysis. The OTC-CNP used in this work was synthesized and characterized as reported in our previous study (6).

### Chemical Reagents

The reagents used were OTC HPLC standard of 98.3% purity (CAS Number 79-57-2) (TargetMol, Boston, USA), phosphoric acid, acetonitrile, and methanol (Fisher Scientific, Malaysia). Ultrapure HPLC water was collected from Milli-Q Integral Water Purification System (type 1) (MilliporeSigma, USA). All other reagents used are of analytical grade.

### Chromatographic Conditions

The plasma concentrations of OTC were measured using a previously described HPLC method (16). This was performed using an isocratic high-performance liquid chromatography system (Agilent Technologies Series 1,200 Autosampler, Agilent Technologies, Wilmington, DE, USA), with a variable-wavelength UV detector (Agilent Technologies 1,200 Series VWD, Agilent Technologies). The OTC in the sample was separated by using a Zorbax stable bond SB C18 column (250 mm × 4.6 mm, 5 μm particle size) at a 1.0 ml/min flow rate. OTC was eluted using mobile phase made up of distilled water, acetonitrile, and methanol (7:2:1); 6.84 g of oxalic acid was added to 1 L of the mobile phase solution. OTC detection was done at 350 nm and column temperature was set at 40°C. The retention time was 4.29 min.

### Preparation of Plasma Samples

Plasma samples were prepared using the method described in Ref. (16) with slight modifications. Briefly, 100 μl of releasing solution consisting of 78% distilled water, 2% phosphoric acid, and 20% acetonitrile was added into 100 μl of plasma. Then, the sample containing plasma and the releasing solution was vortexed for 2 min and filtered using an Ultra-4 centrifugal filter unit (Amicon®). The filtrate was centrifuged at 10,000 rpm at room temperature for 30 min; the clear supernatant was collected into an HPLC injection vial and 50 μl was injected into the HPLC system.

### Method Validation

The correlation coefficient (r) of the linear relationship in the calibration curve was > 0.999 for OTC in plasma across the 10–0.156 μg/ml range. Data for the recovery of OTC in plasma using the HPLC method are presented in Table 1. The accuracy and precision of the method were tested by preparing triplicates samples from 50 to 150% of the target concentrations. The percentage recovery of OTC ranged from 90.10 to 98.40% with percentage relative standard deviation from 0.611 to 1.052%. The limit of quantification and detection was ~ 0.01 and 0.03 ng/ml, respectively.

**Table 1:** Validation data for OTC by high-performance liquid chromatography (HPLC).

**Sample ratio**	**Average % recovery**	**%RSD**	**LOD(ng/ml)**
2:1 (50)	91.30	1.052	0.03
1:1 (100)	90.10	0.295	
1:3 (150)	98.40	0.611	

*n = 5 samples for each concentration used for the analysis*.

### Pharmacokinetic Analysis

The concentrations derived from HPLC analysis were used to calculate the composite pharmacokinetic parameters. Following destructive testing methods, the average plasma concentration at each time point was pooled for each group, and this was used to generate pharmacokinetic parameters by non-compartmental analysis using the PK solver software for pharmacokinetic data analysis “add-on” for Microsoft Excel 2010 (17). Cmax (maximum plasma concentration) and Tmax (time to maximum plasma concentration) were directly obtained from the observed data. The terminal slope (λ_z_) was determined by linear regression of the terminal phase of the log-linear concentration-time profile (using the last three time points). The terminal half-life (T_1/2_λ*z*_) was calculated using the formula 0.693/λ_z_. The AUC was calculated as described in Ref. (18), while the SD of the AUC was calculated using Yuan's method (19) to compare the AUC of the OTC and OTC-CNP groups. Clearance (CL/F) and the apparent volume of the distribution (V_*d*_*/*F) were calculated using the formula: (dose)/*AUC*_(0−∞)_ and (dose)/(λ_z_ × *AUC*_(0−∞)_), respectively (20).

### Statistical Analysis

All results are presented as mean ± SD. Plasma OTC concentrations at each time point were subjected to Student's *t*-test (Graph pad prism version 8.0). Statistical comparisons between the AUC values of OTC and OTC-CNP groups were also determined using unpaired *t-*test (Graph pad prism version 8.0). *P* < 0.05 was considered significant.

## Results

**Figure 1:**
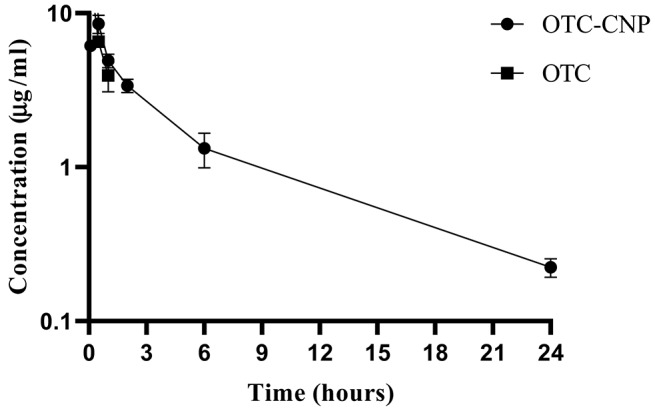
Semilogarithmic (means ± SD) plot of OTC plasma concentration following intraperitoneal (IP) administrations of OTC-CNP and OTC at the dose of 10 mg/kg in *BALB/c* mice (*n* = 5).

IP administration of 10 mg/kg OTC-CNP gave plasma concentrations of OTC quantifiable from 0.083 to 24 h while free OTC administration at the same dosage was detected for up to 1 h only (Figure 1). The plasma concentrations (mean ± SD) of OTC and OTC-CNP across the time points are shown in Table 2. The plasma concentration obtained for OTC at 0.083 h was significantly higher (*P* < 0.05) compared to OTC-CNP. However, at 0.167 to 24 h, concentrations from OTC-CNP was higher than that of free OTC (Table 2).

**Table 2:** Plasma concentration (mean ± SD) of OTC and OTC-CNP in *BALB/c* mice after 10 mg/kg administration.

**Time (h)**	**OTC (μg/ml)**	**OTC-CNP (μg/ml)**
0.00	0	0
0.083	23.53 ± 1.21^***^	6.14 ± 0.14
0.167	12.26 ± 0.42	64.99 ± 2.74^***^
0.25	12.16 ± 0.72	36.73 ± 3.37^**^
0.5	6.53 ± 0.41	8.52 ± 1.14^*^
1	3.94 ± 0.85	4.91 ± 0.49
2	0.00 ± 0.00	3.38 ± 0.33^***^
6	0.00 ± 0.00	1.33 ± 0.29^**^
24	0.00 ± 0.00	0.22 ± 0.03^**^
48	0.00 ± 0.00	0.00 ± 0.00

(*, **, and *** *represent statistical difference between OTC-CNP and OTC at p < 0.05, p < 0.001, and p < 0.0001, respectively)*.

The pharmacokinetic parameters are presented in Table 3. The maximum plasma concentration (C_max_), time to maximum plasma concentration (T_max_), half-life (T_1/2_), mean residence time (MRT), and apparent volume of distribution (V_d_/F) of OTC in the OTC-CNP group were significantly higher (*p* < 0.05) than those of the free OTC. However, the elimination rate constant (K_el_) and the apparent total body clearance (CL/F) were lower in the OTC-CNP treated group (Table 2).

**Table 3:** Pharmacokinetics parameters (mean ± SD) of OTC from non-compartmental analysis after a single dose of 10 mg/kg IP administration of OTC and OTC-CNP in *BALB/c* mice.

**Pharmacokinetic parameter**	**OTC**	**OTC-CNP**
λ_z_ (1/h)	1.163	0.135
T_1/2/_ λ_z_ (h)	0.596	5.133
T_max_ (h)	0.083	0.167
C_max_ (μg/ml)	23.53	64.99
AUC 0 -∞ (μg/ml^*^h)	10.42 (9.43–11.4)	46.68 (40.30–53.07)^*^
MRT 0 -∞ (h)	0.852	4.287
V_d_/F (mg/kg)/(μg/ml)	0.825	1.587
CL/F (mg/kg)/(μg/ml)/h	0.959	0.214

Bailer's and Yuan's method was used to calculate AUC 0-∞ and variance (95% C.I.).

* p = 0.05 (unpaired t-test).

*where λ_z_, terminal slope; T_1/2/_ λ_z_, terminal half-life; T_max_, time to maximum plasma concentration; C_max_, maximum concentration; AUC, area under the curve; MRT, mean residence time; V_d_/F, apparent volume of distribution; CL/F, apparent total body clearance*.

## Discussion

The method developed for the determination of OTC by HPLC was verified based on linearity, recovery, precision, LOQ, and LOD in line with the standard bioanalytical method validation (21). The average percentage recovery of OTC between 90.10 and 98.40% shows that the method developed is accurate and acceptable since the recovery of the analyte from a sample must not necessarily be 100%, but it should be consistent and reproducible (21, 22). The analytical method used in this study is precise as the relative standard deviation (coefficient of variation) is <15% (23). This implies that the analytical method can detect OTC at the stated retention time without interference with other constituents present in the plasma. The LOD and LOQ for OTC suggest that the method is sensitive for detecting OTC, and this agrees with the LOD and LOQ of OTC published earlier (16, 24). The linearity of the calibration curve of the analytical method is excellent with regression coefficient > 0.999, and all the samples measured in this study were above the LOQ.

The pharmacokinetics of OTC in this study was performed using a non-compartmental model (25, 26). Its simplicity, objectivity, and practicability favor its use for description of the time course of drug concentrations in the body (17, 27).

Both drugs were absorbed progressively; free OTC lasted only for an hour while OTC-CNP formulations presented a longer time-plasma profile lasting for up to 24 h.

The fast clearance of OTC disagrees with the findings in Ref. (26) where the absorption of OTC was slow and plasma concentrations lasted for up to 12 h, and this may be because of species differences and the pharmaceutical form of OTC used. On the other hand, the prolonged detection of up to 24 h in the OTC-CNP group indicates slow and sustainable release of OTC from CNP (28). Furthermore, the delivery of antibiotics in nanoparticles is known to cause the sustained release of antibiotics, usually seen as an increase in the half-life of the drug in plasma (29).

The Tmax for free OTC was obtained quickly at 0.083 h. This rapid absorption of free OTC can be explained based on earlier reports where quick absorption of OTC following IP administration in rodents was linked with numerous mesenteric vessels, which allows rapid passage into the bloodstream and after which the blood concentration declines as it distributes to other organs (30). The longer time taken to reach the Tmax of OTC-CNP at 0.167 h may be because of the slow release of OTC from CNP (29). In addition, the absorption of calcium carbonate nanoparticles following administration can be attributed to its size (62.4 ± 20.68 nm) and negative charge (6, 31). At this size, it is easily transported from the peritoneum via the stomata and lymphatic system. Furthermore, the negative charge also facilitates its higher lymphatic vessel uptake rather than being retained in the peritoneum (32).

The significant elongation in T_1/2_ (8.6-fold) with the increase in T_max_ (2-fold), C_max_ (2.8-fold), and AUC (4.5-fold) of OTC-CNP compared to free OTC observed in this study is attributed to the ability of nanoparticles to avoid P-gp-mediated-drug efflux and hepatic first-pass metabolism by cytochrome P450 (CYP450) enzymes (29, 33).

The improved pharmacokinetic parameters of OTC-CNP are an indication that loading OTC into CNP could increase its therapeutic usefulness in diseases caused by intracellular pathogens and biofilm-related infections where maintenance of the antibiotic therapeutic level needs to be sustained for longer period before the next dose is administered (7, 11). Encapsulation of drugs in CNP has been proven to be effective for IP drug delivery (34, 35).

## Conclusion

The study investigated the pharmacokinetics of OTC-CNP and OTC in female *BALB/c* mice at a single dose of 10 mg/kg. The plasma pharmacokinetic parameters of OTC were improved when loaded into CNP. Further studies are necessary to clarify the efficacy and safety of OTC-CNP.

## Data Availability Statement

All datasets generated for this study are included in the article.

## Ethics Statement

The animal study was reviewed and approved by Institutional Animal Care and Use Committee (IACUC), Universiti Putra Malaysia (UPM/IACUC/AUP/R050/2018).

## Author Contributions

SI and AA conceived of the pharmacokinetic study. SI, AA, and MB conducted the experiment. AA, ZA, JA, and S-ZR contributed to sample preparation, data analysis, and general supervision of the project. SI, AA, and ZA wrote the final version of the article with contribution from all other authors. All the authors have read and agreed to the submission of this manuscript to Frontiers in Veterinary Science, Pharmacology, and Toxicology Section.

## Conflict of Interest

The authors declare that the research was conducted in the absence of any commercial or financial relationships that could be construed as a potential conflict of interest.
